# Exploring the Caffeine-Induced Teratogenicity on Neurodevelopment Using Early Chick Embryo

**DOI:** 10.1371/journal.pone.0034278

**Published:** 2012-03-28

**Authors:** Zheng-lai Ma, Yang Qin, Guang Wang, Xiao-di Li, Rong-rong He, Manli Chuai, Hiroshi Kurihara, Xuesong Yang

**Affiliations:** 1 Key Laboratory for Regenerative Medicine of the Ministry of Education, Medical College of Jinan University, Guangzhou, China; 2 Institute of Traditional Chinese Medicine and Natural products, Jinan University, Guangzhou, China; National University of Singapore, Singapore

## Abstract

Caffeine consumption is worldwide. It has been part of our diet for many centuries; indwelled in our foods, drinks, and medicines. It is often perceived as a “legal drug”, and though it is known to have detrimental effects on our health, more specifically, disrupt the normal fetal development following excessive maternal intake, much ambiguity still surrounds the precise mechanisms and consequences of caffeine-induced toxicity. Here, we employed early chick embryos as a developmental model to assess the effects of caffeine on the development of the fetal nervous system. We found that administration of caffeine led to defective neural tube closures and expression of several abnormal morphological phenotypes, which included thickening of the cephalic mesenchymal tissues and scattering of somites. Immunocytochemistry of caffeine-treated embryos using neural crest cell markers also demonstrated uncharacteristic features; HNK1 labeled migratory crest cells exhibited an incontinuous dorsal-ventral migration trajectory, though Pax7 positive cells of the caffeine-treated groups were comparatively similar to the control. Furthermore, the number of neurons expressing neurofilament and the degree of neuronal branching were both significantly reduced following caffeine administration. The extent of these effects was dose-dependent. In conclusion, caffeine exposure can result in malformations of the neural tube and induce other teratogenic effects on neurodevelopment, although the exact mechanism of these effects requires further investigation.

## Introduction

Chick embryos are commonly used in developmental biology studies because of its simplicity and similarity to human embryos. They are also economically efficient and can be easily manipulated *in vitro*. Most importantly, the level of gene expression in avian embryos can be adequately controlled as a result of recent developments in transgenesis techniques [1]. In addition, early chick embryos are reasonably sensitive to external physiochemical compounds such as caffeine. Previous experiments have used chick embryos to test for the effects of caffeine and concluded that caffeine-induced toxicity can lead to teratogenesis and even fetal death. [2].

Caffeine, a white crystalline xanthine alkaloid, was first isolated in the eighteenth century. It was recognized as a stimulant of the central nervous system (CNS) because of its ability to enhance alertness. Other effects of caffeine after ingestion include diuresis, and increased heart rate and or blood pressure [3]. Caffeine was mainly consumed in the Western civilizations. However, it is now distributed worldwide, indwelled in our foods, drinks and medicines [4]–[6]. Its popularity has generated more interest from scientists on the potential harmful effects of caffeine on our health, especially the possibility of abnormal fetal development when ingested by pregnant women. It is estimated that 70–95% of pregnant women from Western civilizations consume 2 cups of coffee everyday. The same amount is taken by a even higher percentage of women of a less educated background [7], [8]. Many animal experiments have studied the effects of caffeine during pregnancy. However, the exact consequence of caffeine on fetal neurodevelopment remains uncertain. This issue is now not only concerned by academics but has gained much public interest as people are becoming more aware of their own health and seek information on such matters.

Development of the nervous system begins with the formation of neural plate, and as the most important event during the developmental process, neurulation consists of three overlapping events in higher vertebrates. First, the neural tube is formed when the dorsal neural folds meet and fuse above the midline of the neural plate. This subsequently gives rise to the CNS. Then, neural crest delamination occurs to give rise to a variety of cell types. This process involves the dorsal-ventral migration of neural crest cells along either side of the neural tube. Cells on each side of the neural tube separate to migrate along both sides of the somites. Finally, the bona fide epidermis is formed. Neurodevelopment is not entirely controlled by spatiotemporal gene expression, but also by the external environment of the embryo, which induces the greatest influence during cranial neural crest cell delamination. Previous studies have shown that caffeine can transfer into the embryo from the external environment [9] and accumulate in the fetal brain [10], [11]. Therefore, it is conceivable that maternal ingestion of caffeine can disrupt the normal processes of neurodevelopment; hence caffeine can act as a teratogen.

In this study, in vivo and in vitro early chick embryos were exposed to caffeine. We assessed the differences between the caffeine-treated embryos and the control in terms of their neurodevelopment morphology, neural crest cell specific marker expression and neuronal differentiation. Moreover, we observed the effects of caffeine on the process of neural tube formation. The results showed caffeine exposure had an effect on the neurodevelopment process of chick embryos. Specifically, the earlier phases of neurulation were significantly altered, which led to the failure of neural tube closure. This defect was most prominent in the prosencephalon regions of the embryo. The later phases of neurulation were also affected in high concentrations of caffeine. Furthermore, caffeine administration induced an abnormal neural crest cell migration pattern.

## Results

### Caffeine administration during early chick embryonic development led to the formation of neural tube defects (NTD)

**Figure 1:**
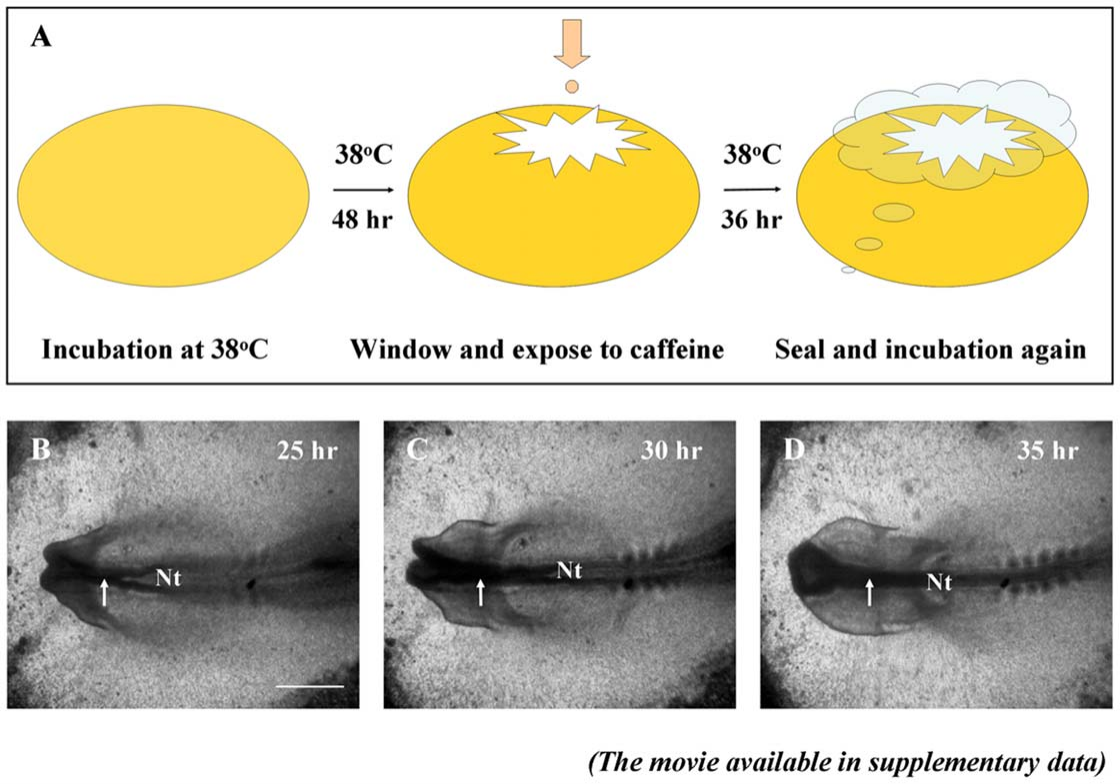
The strategy for administering caffeine to early chick embryo in vivo (A) and normal chick embryo neurulation (B–D). **A:** Schematic drawing of caffeine introduction into early chick embryo *in vivo*. The fertilized egg was windowed on day 1.5, treated with caffeine, then sealed and continually incubated until the required stage. **B–D:** Photographed images of a normal chick embryo development, taken at the incubation 25, 30 and 35 hours. The movie version can be found in the Movie S1. The white arrows indicate the process of neural tube closure. Scale bar = 500 µm in *B–D*. Abbreviation: Nt, neural tube.

**Figure 2:**
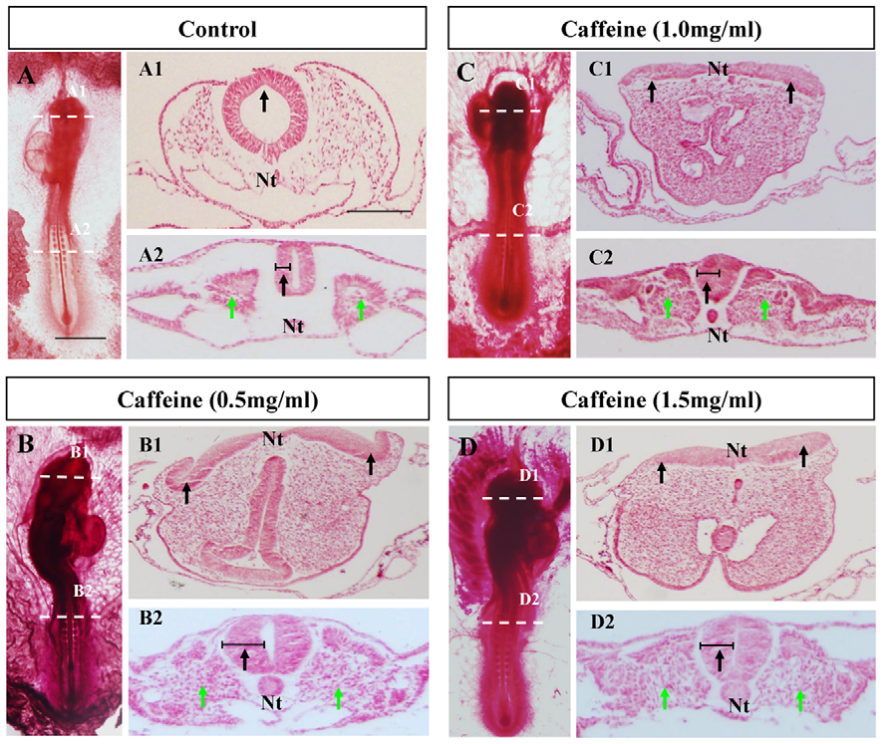
Failure of neural tube closure in the prosencephalon after administering caffeine to chick embryo. **A:** The control embryo was treated with physiological saline solution, and then photographed as a whole-mount embryo and transverse sections at the head and trunk levels, which is indicated by the dotted line (A1 and A2). The black arrowheads indicate the point of neural tube closure, hence neural tube formation. The green arrowheads indicate the development of somites. **B–D:** 0.5, 1 and 1.5 mg/ml caffeine-treated embryos photographed as whole mount embryos. Transverse sections were taken at the head ad trunk levels as indicated by the dotted lines (B1–D2). The transverse sections show the failure of neural tube closure and thickening of the head mesenchymal layer in the prosencephalon (B1–D1), which are indicated by the black arrowheads. Also, the cells of the somites appear more scattered in comparison to the control, as indicated by the green arrows (B2–D2). The process of neurulation is completely inapparent in the high caffeine concentration groups, as indicated by the flatness of the neural plates (C–D). The black horizontal lines on neural tubes (A2–D2) indicate the width of neural tubes. Scale bar = 1 mm in *A–D* and 100 µm in *A1–D2*. Abbreviation: Nt, neural tube.

Studies have demonstrated a link between caffeine consumption and neural tube defects (NTD) [12], [13]. To further investigate this matter, we administered different concentrations of caffeine into *in vivo* chick embryos as shown in Fig. 1A. The NTD number of chick embryos following caffeine exposure was nestled in Table S1 (supporting information). Morphological analysis, aided by Carmine staining (Fig. 2) noted the presence of NTD in the transverse sections of all three concentrations of caffeine-treated embryos (Fig. 2B–D), please note that the NTD are probably not obviously discovered in the whole embryo photograph (Fig. 2B–D) since the magnification or the angle of photographing, more unambiguous NTD phenotype could be observed when transverse sections were accomplished (Fig. 2B1–D1). NTD were not present in the control group (Fig. 2A). Neural tube closure was present in the caudal regions of the embryo in the control group (Fig. 2A1). However, this was not demonstrated in 8 of the 10 each group of caffeine-treated embryos of the same developmental stage as the control, as indicated by the black arrowheads under each neural epithelium (Fig. 2B1–D1). The neural plate of the two higher concentrations of caffeine-treated embryos (Fig. 2C1–D1) remained flat whereas embryos from the 0.5 mg/ml caffeine-treated group showed some signs of neural tube formation as indicated by the black arrowheads (Fig. 2B1). Surprisingly, in addition to the presence of NTD, the prosencephalon sections showed that the cranial mesenchymal tissues were much denser in the caffeine-treated groups (Fig. 2B1–D1) in comparison to the control (Fig. 2A1), though the mechanism of this unknown.

Neural tube closure occurred at the trunk level of the caffeine-treated embryos (Fig. 2B2–D2). But, we found that NTD emerged in the high caffeine concentration group (data not shown). We observed a thickening of the neural tube wall in embryos of which were exposed to a high concentration of caffeine (Fig. 2A2–D2). And in addition to the phenotypes in the nervous system, the somites of the caffeine-treated embryos (Fig. 2B2–D2) appeared less dense in comparison to the control (Fig. 2A2) as indicated by the green arrowheads.

### Migration of HNK1 labeled neural crest cells was disjointed following caffeine treatment, but Pax 7 was relatively unaffected

**Figure 3:**
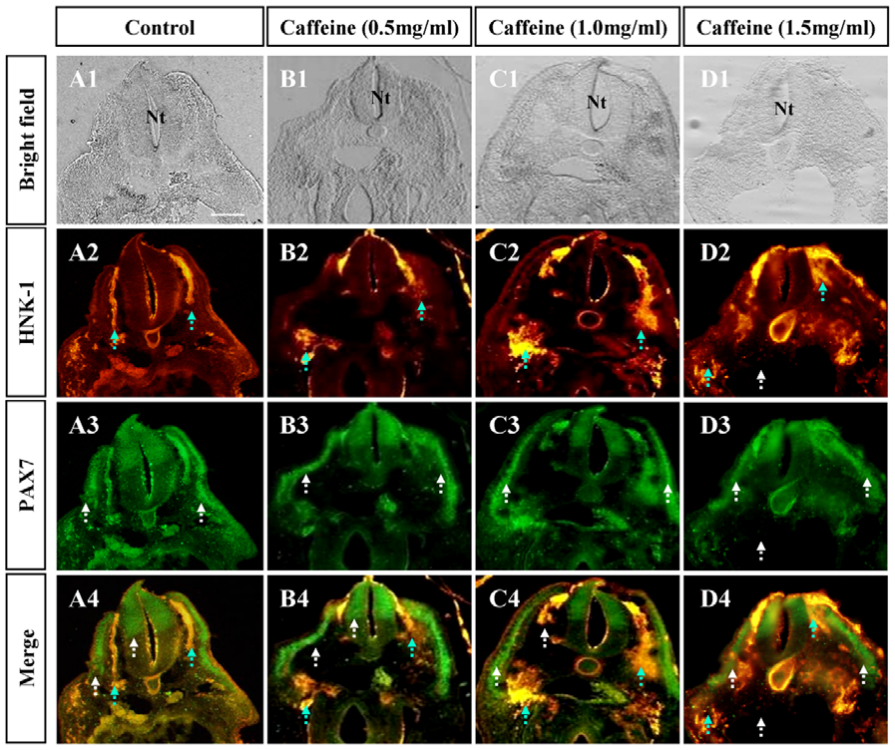
Caffeine induced discontinuity in cranial neural crest cells migration during delamination. **A:** The control embryo, which was treated with physiological saline solution. **B–D:** Caffeine-treated embryos of different concentrations. **A1–D1:** Transverse sections of the control and caffeine-treated embryos at the cranial level. The morphology of the embryos appears normal at caffeine concentrations of under 1.5 mg/ml, above which the structure of the neural tube is damaged (D1). **A2–D2:** Transverse sections of whole-mount immunocytochemistry against HNK1. In the caffeine-treated embryos, the HNK1 labeled migratory neural crest cells showed a disjointed migration trajectory, as indicated by the blue dotted arrows (B2–D2). The normal continuous migration trajectory was demonstrated by the control (A2). **A3–D3:** Transverse sections of whole-mount immunocytochemistry for Pax7. In the caffeine-treated embryos, the Pax7 labeled neural crest cells followed a relatively normal migration trajectory (B3–C3). However, at 1.5 mg/ml concentration of caffeine, there is some structural damage as indicated by the white arrows in D3. **A4–D4:** Images of HNK1 (A2–D2) merged with Pax7 (A3–D3). Scale bar = 100 µm in *A1–D4*. Abbreviation: Nt, neural tube.

Since the caffeine-induced abnormal phenotypes occurred predominantly during neurodevelopment, we decided to focus on a special population of cells called the neural crest cells. These cells originate from the dorsal side of the neural tube and later gives rise to various cell and tissue types, which include neurons, glial cells, adrenal gland cells, the epidermis and connective tissues in the head [14]. Although gene-gene and gene-external environment induced NTD could get through many cellular events, the malformation of neural crest is certainly one of leading suspects. To study the effects of caffeine on neural crest cell migration, i.e. delamination, we used neural crest cell marker Pax7 (dorsal side of neural tube and migratory neural crest cells) [15], [16] and HNK1 (mainly migratory neural crest cells) [17]. The results showed that, at the cranial level, neural tube formation occurred at lower, but not higher caffeine concentrations (Fig. 3D1). The control demonstrated normal neural tube closure (Fig. 3A1). Pax7 was expressed in the control group (Fig. 3A3 and A4) and similarly in the low and middle caffeine concentration groups, but not in the high concentration group due to the morphological alterations outlined previously (Fig. 3B3–D3; B4–D4). Most importantly, the continuity of HNK1 labeled neural crest cells migration between the neural tube and somites was lost following caffeine treatment (Fig. 3B2–D2), possibly due to an increased adherence in this adhesion molecule. Another possibility is the alteration of proliferation in HNK1 positive neural crest cells. The incidence of HNK1-phynotype induced by caffeine-exposure was nestled in Table S1 (supporting information). In order to address the question, we repeated the experiment of caffeine exposure to early developmental embryo, and following immunocytochemistry against HNK1 & PAx7 and DAPI staining. DAPI labeled M-phase nucleus following caffeine exposure were less than control in HNK1 positive cells (Figure S2), which suggest that the inhibition of HNK1 expression following caffeine exposure is partly due to the caffeine-exposed reduced proliferation.

**Figure 4:**
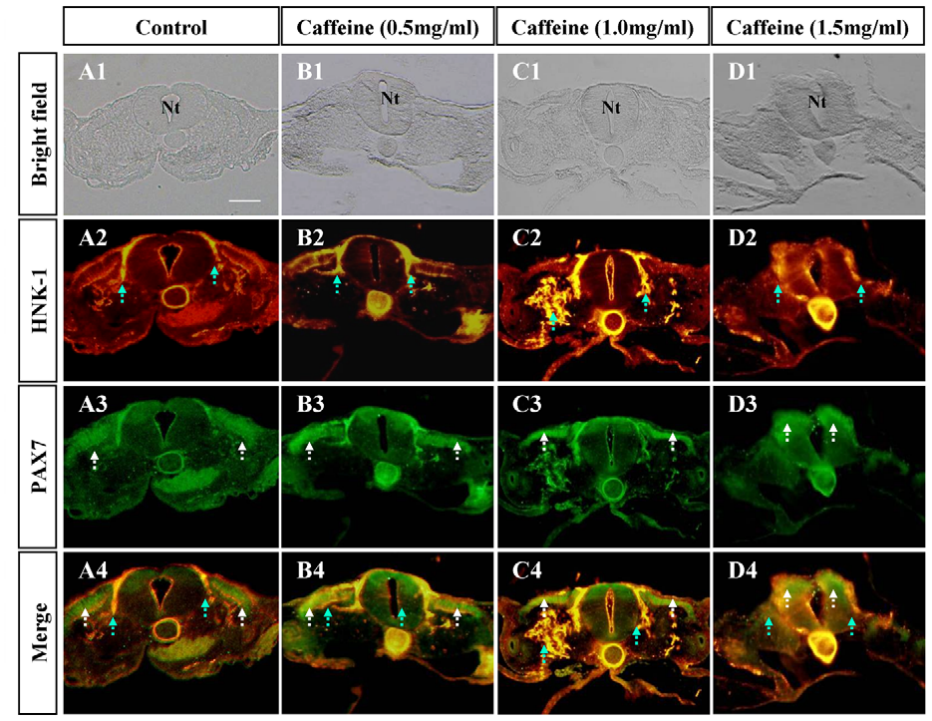
Caffeine induced discontinuity in trunk neural crest cells migration during delamination. **A:** The control embryo, which was treated with physiological saline solution. **B–D:** Caffeine-treated embryos of different concentrations. **A1–D1:** Transverse sections of the control and caffeine-treated embryos at the trunk level. The morphology of the embryos appears normal at caffeine concentrations of under 1.5 mg/ml, above which the structure of the neural tube is damaged (D1). **A2–D2:** A2–D2: Transverse sections of whole-mount immunocytochemistry against HNK1. In the caffeine-treated embryos, the HNK1 labeled migratory neural crest cells showed a disjointed migration trajectory, as indicated by the blue dotted arrows (C2–D2). The normal continuous migration trajectory was demonstrated by the control (A2). **A3–D3:** Transverse sections of whole-mount immunocytochemistry for Pax7. In the caffeine-treated embryos, the Pax7 labeled neural crest cells followed a relatively normal migration trajectory (B3–C3). However, at 1.5 mg/ml concentration of caffeine, there is some structural damage as indicated by the white arrows in D3. **A4–D4:** Images of HNK1 (A2–D2) merged with Pax7 (A3–D3). Scale bar = 100 µm in *A1–D4*. Abbreviation: Nt, neural tube.

At the trunk level, there were an insignificant number of caffeine-treated embryos, which exhibited morphological alternations (data not shown) (Fig. 4A1–D1). Pax7 and HNK1 expression at the trunk level following caffeine administration was relatively similar to the findings at the cranial level (Fig. 4A2–D2; 4A3–D3; A4–D4). HNK1 expressing migratory neural crest cells accumulated at the somite position during its migration (Fig. 4C2 and C4) whilst Pax7 expressing cells followed a very similar pattern to the control (Fig. 4A3–D3; A4–D4). In order to further confirm the irrelevance of caffeine exposure to Pax7 positive cells, we re-photographed HNK1 and Pax7 immunocytochemistry slices with higher magnification in similar experiments of caffeine-exposure (Figure S1). Again, we can see that Caffeine-exposure reduced HNK1 positive migrating neural crest cells (Figure S1A–B) while did not affect the delamination of Pax7 positive cells including neural crest and dermamyotome (Figure S1C–D).

### Caffeine induced suppression of proliferation and differentiation in neural precursor cells

**Figure 5:**
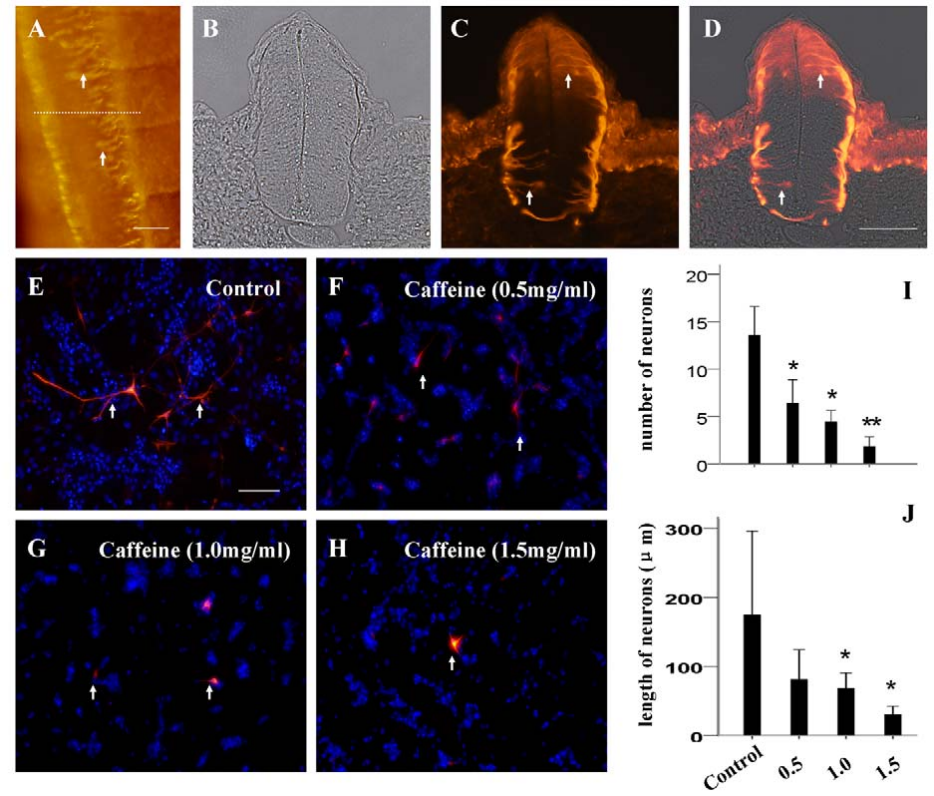
Caffeine induced suppression of neuron proliferation and differentiation. **A–D:** Neurofilament expression pattern of normal developing neural tubes in the whole-mount embryo (A) and transverse sections (B-bright-field; C-fluorescence; D-merge). The white arrowheads indicate neurofilament positive neurons in the neural tube. **E–H:** The disassociated chick embryo brain cells were treated with different concentrations of caffeine for 24 hours. Following which, the number of neurofilament positive cells in the caffeine-treated was reduced in comparison to the control (E,I). Also, the neuron length decreased after caffeine treatment (**J**). Scale bar = 100 µm in *A*, 50 µm in *B–D* and 100 µm in *E–H*.

To further investigate the caffeine-induced impediment on neurodevelopment, we observed the development of neurons in vitro individually following administration of different caffeine concentrations. Within the developing neuron, a specific cytoskeletal element called neurofilament is present. Neurofilament is especially expressed in the axons of the neurons [18]. Hence, neurofilament expression can be used as a marker in the assessment of neuron development following caffeine exposure. First of all, we showed that neurofilament expression is present in the neural tube (Fig. 5A) of normal HH15 chick embryos as indicated by the white arrowheads (Fig. 5C–D). This was most prominent on the dorsal side. In *in vitro* experiment, we carried out primary cell culture in same cell density (2.5×10^5^/ml) of initially disassociated embryonic brain nervous progenitor cells prior to caffeine exposure. *In vitro* culture of normal chick embryonic brain tissue exhibited many neurofilament positive neurons of which had long protrusions (13.6+4.0, n = 7) (Fig. 5E). Caffeine exposure led to a dose-dependent reduction in the number of neurofilament-expressing neurons (6.4±3.2, 4.4+1.6 and 1.9±1.3 respectively, n = 7) (Fig. 5F–H) and the difference between each caffeine-treated group and the control was significant (*p<0.05; **p<0.01) (Fig. 5I). Furthermore, the neuronal length following treatment with middle and high concentrations of caffeine was also significantly reduced (17.3±16.3 µm, 13.6±8.0 µm and 6.1±4.8 µm respectively, n = 16 for each group) (*p<0.05) in comparison to the control (34.9±24.8 µm, n = 16) (Fig. 5J). This suggests that caffeine exposure does lead to impairment of neuron proliferation and differentiation.

## Discussion

The public is constantly reminded of the harmful effects of alcohol and tobacco. Comparatively, less emphasis is placed on adverse effects of caffeine though it is widely consumed as part of our daily intake of foods and beverages. Unfortunately, the general consensus is that caffeine consumption is much safer than alcohol or tobacco.

There is a growing interest to clarify the consequences of caffeine toxicity on fetal development because its consumption is increasing throughout world. Currently, we have a limited understanding of the effects of caffeine on fetal development, which means research is required to improve our appreciation on this matter. To achieve the aim of our study, we employed an embryonic model of which mimicked the human fetal development and subjected this to caffeine treatment. Chick embryos were used because early chick embryonic development is very similar to that of human embryos. Also, they can be easily manipulated in vitro and cultured in vivo following caffeine exposure. In this study, we investigated the potential risks of maternal caffeine-consumption on fetal neurodevelopment by using morphological assessment and immunocytochemistry of neuron and neural crest specific marker expression. In other studies about caffeine exposure induced toxicity to development, 100–600 µM were employed since 100 µM caffeine contraction is the smallest after consuming several cups of coffee or caffeine-containing beverage [19], [20]. However, in our *in vivo* experiment, several times higher concentration of caffeine was utilized. The reason is that the actual concentration of caffeine exposing embryos is probably a bit lower due to the difficulty of diffusion of caffeine in egg.

Using whole embryo cultures exposed to heavy caffeine, Marret et al. found that half of the caffeine-treated embryos demonstrated failure of neural tube closure and excessive proliferation of neuroepithelial cells [20]. In another study using rat embryos, a significantly higher number of neural tubes failed to close in the caudal region of the caffeine-treated embryos (91%) in comparison to the control (14%) [21]. In keeping with these studies, we showed that high concentrations of caffeine exposure led to the formation of chick NTD (Fig. 2). And the embryos that underwent neural tube closure exhibited an increased neural tube thickness at the trunk level, which is similar to the observation in whole mouse embryo culture [20]. A plausible speculation for above phenotype is the impairment of neural fold elevation (Fig. 2B1) during neural tube formation. In addition to the caffeine-induced NTD phenotype, we also found an accumulation of cranial mesenchymal tissue, i.e. it appeared thicker than that of the control. This is possibly as a result of irregular expression of the adhesion molecule HNK1 or the clustering of neural crest cells (Fig. 3 and 4). Interestingly, when Sahir et al. injected pregnant mice intraperitoneally with caffeine (1.25, 2.5, or 5 mg/100 g BW), an acceleration of telencephalic vesicles formation from primitive neuroepithelium evagination was also elicited [22].

Morphologically, neurulation involves neural plate shaping, folding, elevation, closure (refer to Movie S1) and neural crest delamination. Formation of the neural crest is an essential part of neurulation, yet it is often neglected. Neural crest delamination starts at the edge of the neural fold and migrate along 2 paths; one of which migrates above the dermomyotome and later gives rise to melanocytes, while the other migrates below the dermomyotome to give rise to dorsal root ganglia. We labeled the first population of cells with Pax7 and the later with HNK1. These are specific markers for neural crest cells. Interestingly, the migration trajectory of HNK1 labeled neural crest cells was significantly altered following caffeine treatment (Fig. 3 and 4) while Pax7 positive neural crest cell migration appeared comparatively similar to the control. We postulate that these findings are the result of alterations in neural tube gene expression following caffeine exposure. Therefore, by further investigation of neural tube gene expression, we hope to find the mechanism behind the caffeine-induced effects outlined above. In relation to this, Shh may be involved as it is overly-expressed in both cultured neurons and astrocytes following caffeine exposure [23].

NTD could be considered as the failure of neural fold elevation. In Splotch (Sp) and splotch-delayed (Spd) mice, which have neural tube defect, there simultaneously are structural deficiencies derived in neural crest cells as well [24]. It was reported that the cranial NTD was to due to the alteration of cranial neural crest cells in *shrm* mutation mice [25]. The cranial NTD often accompany the impairment of neural crest cell migration, in which the causation of facial clefting and cardiac defects is plausibly abnormal neural crest migration subsequently malformation of the face and cardiovascular structures [26]. Taken together, it is obviously that neural tube defect is relevant to the malformation of neural crest derivation. It could be due to abnormal migration of neural crest cells following caffeine exposure (Fig. 3B_D), of course, we can't eliminate the possibility that it was because of the disturbance of proliferation of those neural crest cells on the way to their derivative determinations. We also observed that the proliferation in HNK1 positive neural crest cells was inhibited to some extent following caffeine exposure (Figure S2). In addition, abnormal apoptosis in neural tube could result in NTD too.

Neuron development and differentiation plays an essential role in neurodevelopment. Using Immunohistochemistry, we showed that neurofilament is expressed by developing neurons of the neural tube, in particularly on the dorsal side (Fig. 5A–D). This warranted our used of neurofilament expression to assess the development and differentiation of neurons in cultured brain cells *in vitro* with or without caffeine. The number of neurofilament positive cells was drastically reduced following caffeine treatment of all three concentrations (Fig. 5E–H). Also, the length of the projections was significantly shorter in the caffeine-treated groups in comparison to the control (Fig I–J). These findings suggest large quantities of caffeine exposure inhibit neuron proliferation and differentiation.

However, despite the findings on abnormal embryonic development following caffeine exposure, the current experimental data is not sufficient to label caffeine as a teratogen. Nonetheless, we should inform the public to be cautious of caffeine consumption during pregnancy and recommend the avoidance of large quantities of caffeine ingestion if possible.

## Materials and Methods

### Chick embryo incubation and caffeine exposure

The fertilized leghorn eggs were obtained from the Avian Farm of the South China Agriculture University, then incubated in a humidified incubator (Yiheng Instruments, Shanghai, China) at 38°C. Once the embryos reached the HH10 stage (Hamburger and Hamilton [27]), which generally required 48 hours of incubation, caffeine (Nacakai, Japan) was administered using the technique shown in Fig. 1A. The caffeine-treated embryos were then incubated for a further 36 hours before they were fixed with 4% paraformaldehyde.

### Primary culture for chicken embryonic brain cells

The head tissue of the chick embryos after 11 days of incubation were cut into pieces, washed with PBS and digested by immersing the tissues in trypsase for 10 minutes, repeated centrifuging and suspending in DMEM-12 (GIBCO) for three times, finally the brain cells were cultured *in vitro* in culture medium (DMEM-F12) in an incubator (Galaxy S, RS Biotech) at 37°C and 5% CO_2_ for a duration of 24 hours.

### Immunohistochemistry

Immunohistochemistry was performed on the whole-mount control and caffeine-treated chicken embryos to detect HNK1 (N-CAM, IgM), Pax7 and neurofilament protein expression as described by Yang X et al [28]. In brief, chick embryos were fixed with 4% paraformaldehyde (PFA) at 4°C overnight. The unspecific immunoreactions were blocked with a solution containing 2% Bovine Serum Albumin (BSA), 1% Triton-X and 1% Tween 20 in PBS for 2 hours at room temperature. The embryos were then washed using PBS and incubated overnight at 4°C on a rocker with primary monoclonal antibody mixtures raised against HNK1 (sigma, 0.002 ug/ul), Pax7 (DSHB, 1∶100) or neurofilament (Invitrogen, 1∶200). After a further, more extensive wash with PBS, the embryos were incubated overnight at 4°C on a rocker once again with specific secondary antibody mixtures coupled with Alexa Fluor 555, 488 or both (Invitrogen, 0.002 mg/ml), to visualize the primary antibodies.

### Acetic Carmine Staining

Acetic carmine staining (Shanghai Zhanyu, Ltd) of whole-early chick embryo was prepared by adding 5 g carmine to 200 ml 50% acetic acid, then placed in a boiling water bath for 15 minutes prior to filtering. The whole mount-chick embryos were treated with the acetic carmine stain overnight, then washed in distilled water for several minutes. To better visualize the structural details of the chick embryo, the embryos were transferred into a 1% hydrochloric acid in 70% ethanol solution, followed by glycerin, which both reduced the colorization of the staining.

### Photography

Following immunohistochemistry, the whole mount embryos were photographed using a stereo-fluorescence microscope (Olympus MVX10) and processed by the Olympus software package Image-Pro Plus 7.0. The embryos were then sectioned into 15 µm thick slices using a cryostat microtome (Leica CM1900), photographed using an epi-fluorescent microscope (Olympus IX51, Leica DM 4000B) at a magnification of 200× and 400×, and processed using the CW4000 FISH Olympus software package. The time-lapse movie was made by using Olympus software, from bright-field images of developing chick embryos in a microscope incubator taken at 3-minute intervals.

### Statistical analysis

The data were presented as mean ± SE. The statistical analysis for the experimental data was performed using the SPSS 13.0 statistical package program for windows. Normal distribution data were subjected to a paired t-test. P<0.05 was considered to be the level of significance.

## Supporting Information

Movie S1 **The process of neural tube closure during early chick embryo development.** Bright-field images of a HH7 chick embryo were continuously taken at 3-minute intervals in an inverted microscope while it was developing in the microscope incubator. Using the VideoMach software, the movie was made of the continuous bright-field images, in which we can clearly observe the fusion process of bilateral edges of neural plate at midline during neural tube formation.
(AVI)

Figure S1 **Caffeine-exposure is irrelevant to Pax7 positive cells although reducing HNK1 positive neural crest cells.** (**A–B**) the immunocytochemistry against HNK1 for control (A) and caffeine-exposed (0.5 mg/ml) (B) embryos, in which Caffeine-exposure reduced HNK1 positive migrating neural crest cells (B) compared to control (A). (**C–D**) the immunocytochemistry against Pax7 for control (C) and caffeine-exposed (0.5 mg/ml) (D) embryos, in which PAX7 is visibly expressed in dorsal neural tube & pre-migratory neural crest cells (white arrowheads) and dermamyotome (red arrowheads). No altered delamination of *Pax7* positive cells including neural crest and dermamyotome (S2C–D) was found following Caffeine-exposure. Scale bar = 100 µm in *A–D*. Abbreviation: Nt, neural tube.
(TIF)

Figure S2 **Caffeine-exposure reduced the proliferation of HNK1 positive migratory neural crest cells.** (**A1–B1**) the transverse sections of immunocytochemistry against HNK1 for control (A1) and caffeine-exposed (1.0 mg/ml) (B1) embryos respectively. (**A2–B2**) the transverse sections of immunocytochemistry against HNK1 (red) and Pax7 (green) for control (A2) and caffeine-exposed (1.0 mg/ml) (B2) embryos respectively. (**A3–B3**) DAPI staining for control (A3) and caffeine-exposed (1.0 mg/ml) (B3) embryos respectively. (**C**) high magnification of DAPI staining M-phase nucleus (white arrowheads) in control indicated by triangle in A3. (**D**) high magnification of DAPI staining M-phase nucleus (white arrowheads) in caffeine-exposure indicated by triangle in B3. (**E**) Statistical chart for the number of control and caffeine exposure. Scale bar = 100 µm in *A–B* and 10 µm in *C–D*.
(TIF)

Table S1 **The survey for number of neural tube defect embryos and inhibitive incidence of HNK1 positive neural cells induced by caffeine-exposure.** The upper Table represents the NTD embryo number following the administration of three concentrations of caffeine. There was a dose-dependent manner between the incidence of NTD and caffeine-administration concentrations. The lower Table represents the incidence of HNK1-expression abnormality following the administration of caffeine, which also became bigger along with the caffeine-concentration increase.
(DOC)
