# Correction: Exploring the Caffeine-Induced Teratogenicity on Neurodevelopment Using Early Chick Embryo

**DOI:** 10.1371/annotation/0dc7c4d1-2601-4267-b56d-a315d89a6310

**Published:** 2012-08-09

**Authors:** Zheng-lai Ma, Yang Qin, Guang Wang, Xiao-di Li, Rong-rong He, Manli Chuai, Hiroshi Kurihara, Xuesong Yang

The third author, Guang Wang, should be noted as contributing equally to this work along with the first and second authors, Zheng-lai Ma and Yang Qin. 

