# Supplementary figures and images for: Exploring the Caffeine-Induced Teratogenicity on Neurodevelopment Using Early Chick Embryo
Source: PLoS One. 2012 Mar 28;7(3):e34278. doi: 10.1371/journal.pone.0034278 (PMC3314624; doi:10.1371/journal.pone.0034278)

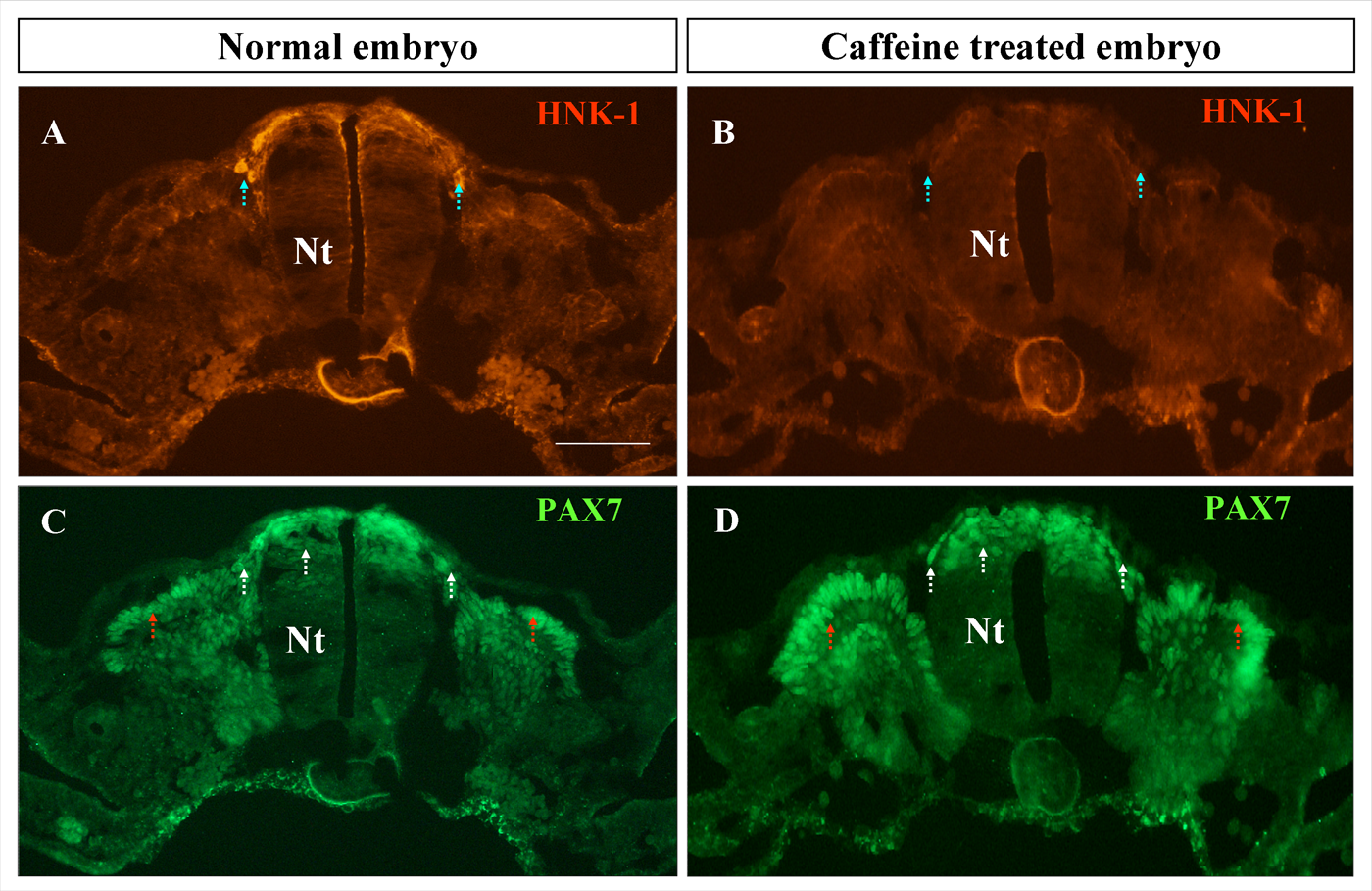

Supplement: Figure S1 — Caffeine-exposure is irrelevant to Pax7 positive cells although reducing HNK1 positive neural crest cells. (A–B) the immunocytochemistry against HNK1 for control (A) and caffeine-exposed (0.5 mg/ml) (B) embryos, in which Caffeine-exposure reduced HNK1 positive migrating neural crest cells (B) compared to control (A). (C–D) the immunocytochemistry against Pax7 for control (C) and caffeine-exposed (0.5 mg/ml) (D) embryos, in which PAX7 is visibly expressed in dorsal neural tube & pre-migratory neural crest cells (white arrowheads) and dermamyotome (red arrowheads). No altered delamination of Pax7 positive cells including neural crest and dermamyotome (S2C–D) was found following Caffeine-exposure. Scale bar = 100 µm in A–D. Abbreviation: Nt, neural tube. (TIF) [file pone.0034278.s002.tif]

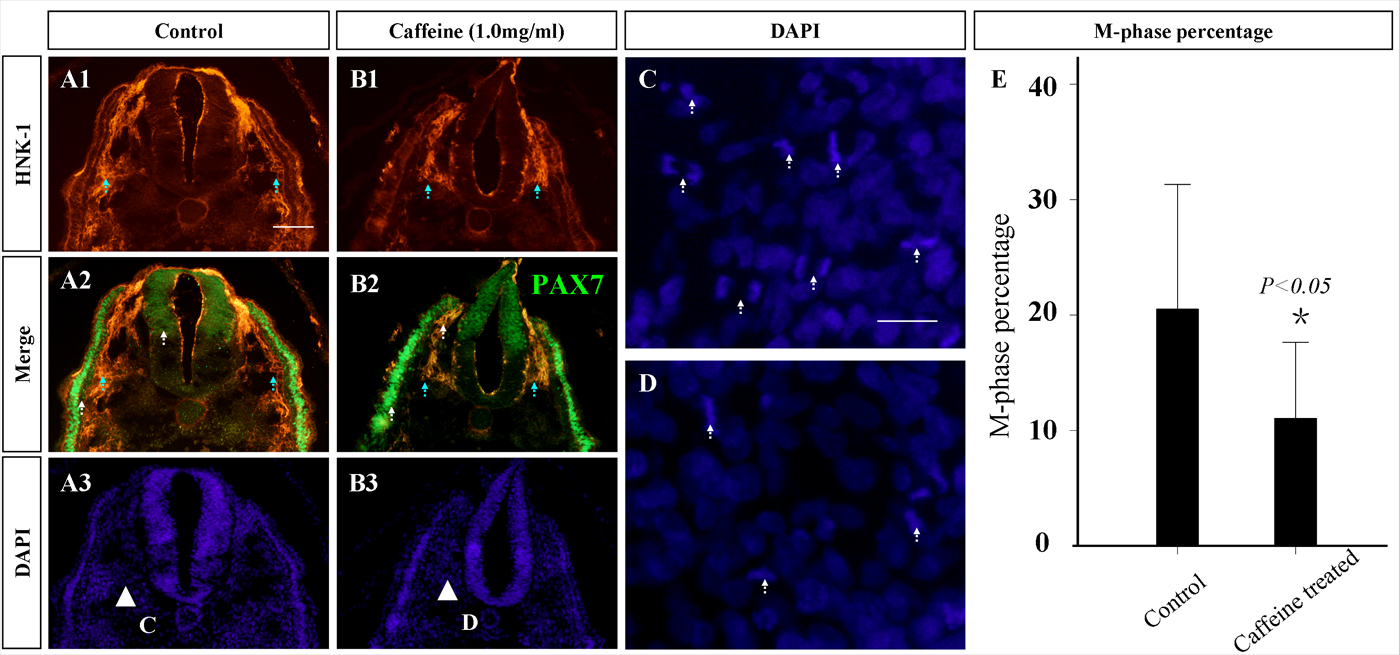

Supplement: Figure S2 — Caffeine-exposure reduced the proliferation of HNK1 positive migratory neural crest cells. (A1–B1) the transverse sections of immunocytochemistry against HNK1 for control (A1) and caffeine-exposed (1.0 mg/ml) (B1) embryos respectively. (A2–B2) the transverse sections of immunocytochemistry against HNK1 (red) and Pax7 (green) for control (A2) and caffeine-exposed (1.0 mg/ml) (B2) embryos respectively. (A3–B3) DAPI staining for control (A3) and caffeine-exposed (1.0 mg/ml) (B3) embryos respectively. (C) high magnification of DAPI staining M-phase nucleus (white arrowheads) in control indicated by triangle in A3. (D) high magnification of DAPI staining M-phase nucleus (white arrowheads) in caffeine-exposure indicated by triangle in B3. (E) Statistical chart for the number of control and caffeine exposure. Scale bar = 100 µm in A–B and 10 µm in C–D. (TIF) [file pone.0034278.s003.tif]
