# Supplementary material for: Exploring the Caffeine-Induced Teratogenicity on Neurodevelopment Using Early Chick Embryo
Source: PLoS One. 2012 Mar 28;7(3):e34278. doi: 10.1371/journal.pone.0034278 (PMC3314624; doi:10.1371/journal.pone.0034278)
Supplement: Table S1 — The survey for number of neural tube defect embryos and inhibitive incidence of HNK1 positive neural cells induced by caffeine-exposure. The upper Table represents the NTD embryo number following the administration of three concentrations of caffeine. There was a dose-dependent manner between the incidence of NTD and caffeine-administration concentrations. The lower Table represents the incidence of HNK1-expression abnormality following the administration of caffeine, which also became bigger along with the caffeine-concentration increase. (DOC) [file pone.0034278.s004.doc]

Caffeine exposure induced neural tube defect (NTD) of chick embryos

| **Caffeine dose (mg/ml)** | **Number of embryos** | **Number of NTD** | **Malformations Percentage (%)** |
| --- | --- | --- | --- |
| 0 | 10 | 0 | 0 |
| 0.5 | 10 | 1 | 10^NS^ |
| 1.0 | 10 | 6 | 60^a^ |
| 1.5 | 10 | 7 | 70^a^ |

One-way ANOVA test .NS , non-significant difference; ^a^*P<0.05 .*

The incidence of HNK-1 expression abnormality following caffeine exposure

| **Caffeine dose**  **(mg/ml)** | **Number of total HNK-1 expression sites** | **Number of HNK-1**  **Expression abnormality** | **HNK-1 expression abnormality percentage (%)** |
| --- | --- | --- | --- |
| 0 | 30 | 0 | 0 |
| 0.5 | 30 | 2 | 6.7 ^a^ |
| 1.0 | 30 | 17 | 56.7 ^b^ |
| 1.5 | 30 | 23 | 76.7 ^b^ |

One-way ANOVA test . ^a^*P<0.05; b ^b^P<0.01*
